# Metabolomics-based analysis of the effects of different storage temperatures on the post-harvest quality of truffles

**DOI:** 10.3389/fpls.2026.1880094

**Published:** 2026-07-23

**Authors:** Yuan Qing, Junhong Jiang, Yao Wang, Qiang Li, Deju Yang, Siying Wu, Xiaoyin Dan, Hai Li

**Affiliations:** 1 College of Agricultural Sciences, Xichang University, Xichang, China; 2 Panxi Crop Improvement Key Laboratory of Sichuan Province, Xichang University, Xichang, China; 3 School of Food and Biological Engineering, Chengdu University, Chengdu, China

**Keywords:** low temperature, postharvest, preservation, quality, truffle

## Abstract

To investigate the effects and mechanisms of different low-temperature storage conditions on the postharvest quality of truffles. This study used truffles from Huidong County, Sichuan Province as the research subject, setting four storage temperatures: -4 °C, 0 °C, 4 °C, and 20 °C. Physiological indicators such as postharvest decay rate, relative electrical conductivity, and respiration rate were measured. These were combined with metabolomic analysis to screen for differential metabolites and metabolic pathways. The results showed that truffles stored at -4 °C have a relatively low rot rate and are better suited for storage(*P* < 0.05). However, the relative electrical conductivity and MDA content of truffles stored at -4 °C were significantly higher than those in the 0 °C and 4 °C treatment groups (*P* < 0.05). The SDH activity of truffles stored at 0 °C and 4 °C was significantly higher than that of the -4 °C group in the later stages (*P* < 0.05). CAT activity was significantly higher in the -4 °C group than in the other groups during the first 21 days of storage(*P* < 0.05). Metabolomic analysis revealed that the -4 °C vs 4 °C group had the most differential metabolites in the positive ion mode, with pathways such as branched-chain amino acid metabolism, glutathione metabolism, and steroid biosynthesis being significantly enriched (*P* < 0.05). This study systematically reveals the effects of storage temperature on truffle preservation and its mechanisms, providing a theoretical basis for postharvest preservation of truffles.

## 1 Introduction

Truffles are ascomycete fungi, also known as “Songlu” in Chinese. Due to their characteristics resembling Poria cocos, they are also called “Tu Fuling”. They belong to the class Ascomycetes, order Pezizales, family Tuberaceae, and genus Tuber. They are characterized by their ectomycorrhizal subterranean fruiting bodies (Herrero de Aza et al., 2022; Angeloni et al., 2025). The fruiting bodies contain a substance similar to boar androgens, α-androstenol, which attracts sows to root and consume them, hence the name “Zhugongjun” in the Yunnan and Panxi regions of China. Truffles have a low natural yield and hold a high economic value in the edible mushroom market, being listed alongside caviar and foie gras as the “Three Western Delicacies. In addition to abundant nutrients and proteins, truffles contain polysaccharides, flavonoids, alkaloids, and other bioactive compounds beneficial to human health (Wang and Marcone, 2011; Adeeyo et al., 2026).

During postharvest storage of truffles, the respiratory metabolism of the fruiting bodies is relatively vigorous (Rivera et al., 2010), and surface microorganisms can easily proliferate in large numbers, leading to decay and spoilage (Tejedor-Calvo et al., 2024). This severely affects the commodity’s shelf life and economic value (Roohinejad et al., 2017). During storage, temperature is the primary influencing factor (Mataragas et al., 2006). Low temperatures can inhibit cellular respiration rates (Price and Sowers, 2004), delay metabolic processes, and also suppress the proliferation of harmful microorganisms to some extent, making it one of the common methods for food and fruit preservation (Chaudhary et al., 2025). However, unsuitable low temperatures may cause chilling injury, leading to increased electrolyte leakage (Khedr et al., 2025), accumulation of reactive oxygen species (Zhou et al., 2025), and accelerated tissue senescence (Yuan et al., 2024). Current research on low-temperature storage of truffles has mostly focused on single temperatures, while there is still a lack of in-depth investigation into the changes and mechanisms of truffles under different storage temperatures. Previous studies have found that storage temperatures close to freezing can significantly preserve food quality, extend shelf life, and maintain nutritional value (Bilbao-Sainz et al., 2021; Lacombe et al., 2025; Maida et al., 2025; Năstase et al., 2026). Zhang et al. (2024) found the effects of cold storage duration on the expression of genes related to oxidative metabolism in Chinese black truffles, indicating that truffle fruiting bodies maintain low metabolic activity during cold storage. Abrashev et al. (2025) reported that low temperatures lead to a decrease in microbial metabolic activity, reduced ATP utilization, the accumulation of electrons at specific points in the respiratory chain, and a rapid increase in ROS production. Currently, research on the low-temperature preservation of truffles has largely focused on the effects of a specific refrigeration temperature (such as 4 °C) on quality maintenance, while systematic comparisons of the differences in truffle physiological responses under various low-temperature gradients (particularly near-freezing temperatures) remain scarce. Xie et al. (2025). believe that metabolomics has been used to identify post-harvest quality biomarkers and to characterize the mechanisms underlying flavor development and quality deterioration.

More importantly, there are currently two distinct knowledge gaps that remain unresolved: First, although near-freezing storage (e.g., -4 °C) can significantly inhibit microbial spoilage, it is unclear whether it induces irreversible damage to truffle cell membranes (cold damage) due to excessive low-temperature stress, and the physiological trade-off mechanism between “microbial inhibition for preservation” and “cold damage” in truffles remains unclear; Second, there is a lack of in-depth molecular analysis at the metabolic network level regarding the synergistic or antagonistic relationships among intracellular energy metabolism (the TCA cycle), antioxidant defense systems, and membrane lipid metabolism in truffles under different low-temperature conditions (–4 °C, 0 °C, 4 °C).

Currently, research on the low-temperature preservation of truffles has largely focused on the effects of a specific refrigeration temperature (such as 4 °C) on quality maintenance, while systematic comparisons of the differences in truffle physiological responses under various low-temperature gradients (particularly near-freezing temperatures) remain scarce. Although near-freezing storage (e.g., -4 °C) can significantly inhibit microbial spoilage, it remains unclear whether such conditions induce irreversible damage to truffle cell membranes (cold damage) due to excessive low-temperature stress, and what physiological trade-off mechanisms truffles employ between “microbial inhibition for preservation” and “cold damage”; Furthermore, there is a lack of in-depth molecular analysis at the metabolic network level regarding the synergistic or antagonistic relationships among intracellular energy metabolism (the TCA cycle), antioxidant defense systems, and membrane lipid metabolism in truffles under different low-temperature conditions (–4 °C, 0 °C, 4 °C). This study uses truffles from Huidong County, Sichuan Province as the research subject, setting four storage temperatures: -4 °C, 0 °C, 4 °C, and 20 °C. By measuring changes in physiological indicators such as postharvest decay rate, relative electrical conductivity, respiration rate, tissue microstructure, MDA content, SDH and CAT activity, and combining metabolomic analysis to screen differential metabolites and enrich related metabolic pathways, this study systematically reveals the effects of storage temperature on truffle storage and its mechanisms, providing a theoretical basis for postharvest preservation of truffles.

## 2 Materials and methods

### 2.1 Experimental materials

Fresh *Tuber indicum* produced in Huidong County, Liangshan Yi Autonomous Prefecture, Sichuan Province were selected. After washing off the surface sediment with clean water, they were rinsed with sterile distilled water, spread on sterile paper, and air-dried in a ventilated area for later use. Before the experiment, the maturity of the purchased truffle samples needed to be assessed. The evaluation method was as follows: Thin slices of truffles were observed under a microscope to check spore color. Three random samples were observed, and if all appeared dark brown or brown, the maturity was considered to be >80%. From the qualified truffle samples with acceptable maturity, fresh truffles of uniform size (approximately 3 cm in diameter) and undamaged surfaces were randomly selected. Commercial PE flat-mouth fresh-keeping bags (polyethylene packaging bags, specifications: 38 cm × 25 cm × 0.0007 cm, temperature resistance: -30 °C to 110 °C) were used for packaging and labeled as Day 0. Each bag contained (500 ± 10) g of samples, which were stored at -4 °C, 0 °C, 4 °C, and 20 °C, respectively. Samples were taken every 7 days for analysis. One portion of the sampled truffles was used for physical index measurements on the same day, while another portion was flash-frozen with liquid nitrogen to preserve enzyme activity. It was then placed in pre-prepared homogenization bags labeled with dates, batches, and other markings, and stored in a -80 °C ultra-low temperature freezer.

### 2.2 Experimental design

This experiment employed a completely randomized design. Four storage temperature treatments were established: -4 °C, 0 °C, 4 °C, and 20 °C (room temperature control). Each temperature treatment included 3 biological replicates, with each replicate consisting of 1 PE storage bag (dimensions: 38 cm × 25 cm × 0.0007 cm). Each bag contained (500 ± 10) g of mature truffle fruiting bodies of uniform size (approximately 3 cm in diameter) and with undamaged surfaces. All treatments were simultaneously placed in a constant-temperature incubator (± 0.5 °C) at the corresponding temperature for a storage period of 35 days. Sampling and analysis were conducted on days 0, 7, 14, 21, 28, and 35 of storage. Day 0 consisted of unstored initial samples, serving as the common starting point for all treatments.

At each sampling, an appropriate amount of fruiting bodies was randomly selected from each replicate bag and divided into three portions: the first portion was used for same-day determination of decay rate, relative electrical conductivity, respiration rate, and fixation of samples for microscopic structure (SEM) analysis; the second portion was flash-frozen in liquid nitrogen and transferred to a -80 °C ultra-low temperature freezer for subsequent determination of MDA content, SDH, and CAT enzyme activities; the third portion was also flash-frozen in liquid nitrogen and stored for metabolomics analysis (samples were collected only on day 28; since the decay rate reached 100% by day 28 in the 20 °C treatment, metabolomics analysis was limited to the three low-temperature treatments at −4 °C, 0 °C, and 4 °C, with pairwise comparisons). All parameter measurements treated each biological replicate as an independent sample, with three technical replicates performed within each replicate. The final data are expressed as the mean ± standard error of the three biological replicates.

### 2.3 Decay rate

Each sample (500 ± 10) g, the diameter of the truffle fruiting body is about 3 cm, and the specific quantity is subject to actual conditions. The observation method is used for determination, mainly checking whether there are mold spots on the surface of the truffle and soft areas with a putrid odor and sticky regions (Zhao et al., 2013). A truffle fruiting body with mold spots or putrid sticky spots larger than 0.5 cm in diameter is counted as rotten. The decay rate of truffles is calculated using the following formula:


$$\text{W}\;(\%)=\frac{F0}{F1}\times 100\%$$

Where: W is the decay rate, F0 is the number of rotten truffles, and F1 is the initial total number of truffle fruits.

### 2.4 Relative electrical conductivity

Slice the truffle fruiting bodies into thin slices, rinse them three times with deionized water, and blot dry with filter paper. Soak the fruiting body slices in 25 mL of distilled water for 1–3 hours. Measure the conductivity of the resulting truffle test solution using a conductivity meter. Boil the solution containing the truffle slices for 15 minutes, allow it to cool to room temperature, and then measure the conductivity of the solution again using a conductivity meter (Zhao et al., 2021).


$$\text{Relative Electrical Conductivity}={\text{C}}_{1}/{\text{C}}_{2}\times 100\%$$

Where: C1: Electrical conductivity of the truffle solution measured after soaking in distilled water, μs/cm; C2: Electrical conductivity of the truffle solution measured after boiling and cooling, μs/cm.

### 2.5 Respiration rate

The static method (Iqbal et al., 2009; Ozturk and Aktas, 2022) was used for measurement in a non-ventilated indoor environment. Truffles were placed in a sealed assimilation chamber (a cube with 10 cm sides), and the carbon dioxide produced by their respiration was collected and connected to a portable photosynthesis system. The carbon dioxide concentration in the assimilation chamber was measured every 2 seconds. The respiration rate was calculated using the following formula:


Respiration rate mg/kg·h=60×(y−x)×t×1/m

Where: x is the CO_2_ concentration value measured in the assimilation chamber at 30 seconds; y is the CO_2_ concentration value measured in the assimilation chamber at 180 seconds; t is the time interval between the first and last measurements; m is the mass of truffles used for determining the respiration rate, in grams.

### 2.6 SEM

Slice the truffle fruiting bodies under different storage conditions into thin sections and soak them in a phosphate buffer solution (pH 7.2) containing 2.5% glutaraldehyde for 24 hours. After soaking, dehydrate the truffle tissue sections sequentially with 30%, 50%, 70%, 80%, 90%, 95%, and 100% ethanol solutions, followed by vacuum freeze-drying for 24 hours. Store the samples in food-grade sterile bags with desiccants at low temperatures for later use. During testing, the freeze-dried truffle slices are coated with gold using a sputter coater to ensure conductivity (Xie et al., 2025), and then observed for tissue structure using a scanning electron microscope (Guo et al., 2023).

### 2.7 Determination of malondialdehyde content

The determination was conducted using the thiobarbituric acid method (Jiang et al., 2020). Different storage conditions of ultra-low temperature frozen truffle samples were weighed, with 0.2 g each placed into 2.0 mL microcentrifuge tubes (EP tubes). Then, 1.0 mL of TCA solution was added and homogenized by grinding. The mixture was centrifuged in a low-temperature centrifuge under the following conditions: temperature set to 4 °C, speed at 10,000×g for 20 minutes. The supernatant was collected into 1.5 mL EP tubes for later use. During the measurement, 0.7 mL of the supernatant was transferred to a 96-well plate, followed by adding 0.7 mL of a 0.67% TBA solution. After mixing, the solution was incubated in boiling water for 20 minutes. After cooling to room temperature, it was centrifuged again (same parameters as above), and the supernatant was transferred to 1.5 mL EP tubes. Then, 200 μL was pipetted into the 96-well plate to measure the absorbance values of the sample solution at wavelengths of 450 nm (λ_1_), 532 nm (λ_2_), and 600 nm (λ_3_) (Kong et al., 2025). The following formula was used for calculation:


$$\text{c}\;(\text{μmol}/\text{L})=6.45\times ({\text{OD}}_{532}-{\text{OD}}_{600})-0.56\times {\text{OD}}_{450}$$

In the formula: OD_450_, OD_532_, and OD_600_ represent the absorbance values of the sample solution at wavelengths of 450 nm, 532 nm, and 600 nm, respectively.

After calculating the error caused by sucrose using the above formula, the MDA content per gram of truffle sample is determined according to the following formula, expressed in nmol/g.


$$\text{SMDA}=\frac{c\times V}{\text{Vs}\times m\times 1000}\times 1000\;(\text{nmol}/\text{g})$$

In the formula: c: Malondialdehyde concentration in the test sample mixture, μmol/L; V: Total volume of the sample extraction solution, mL; Vs: Volume of the sample solution taken for measurement, mL;m: Mass of the truffle sample used for testing, g.

### 2.8 Metabolic enzyme activity assay

The metabolic enzyme activities in the samples were analyzed using commercially available detection kits to assess enzyme activity in truffles under different storage days and temperatures, including CAT (Solarbio Science & Technology Co., Ltd., Beijing, China) and SDH (Solarbio Science & Technology Co., Ltd., Beijing, China). The testing methods followed the kit instructions.

### 2.9 Metabolomics

Based on the physiological quality analysis of stored truffles under different temperature conditions, samples of low-temperature stored truffles at 28 days were selected for metabolomic analysis. 1 g of the sample was freeze-dried, and 100 μL of an 80% methanol aqueous solution was added. The mixture was vortexed and then placed in an ice bath for 5 minutes. After that, it was centrifuged at 15000 g and 4 °C for 15 minutes. A certain amount of the supernatant was taken and diluted with mass spectrometry-grade water to a methanol content of 53%. The resulting solution was then centrifuged at 15000 g and 4 °C for 15 minutes, and the supernatant was collected and injected into LC-MS for analysis.

To monitor the reproducibility of the analytical process and the stability of the instrument, an equal volume of supernatant is drawn from each test sample and mixed to prepare a composite quality control (QC) sample. The QC samples were interspersed in the sample sequence at the same intervals as the test samples (one QC sample inserted for every six test samples). At the same time, an 80% methanol-water solution containing no biological samples (but containing the same internal standard) was used as the process blank control, prepared according to the same pretreatment procedure, to account for background noise and solvent interference. To correct for systematic errors arising during sample preparation and instrument operation, the sum normalization method was used to normalize the peak areas of each sample. Specifically, the peak area of each metabolite was divided by the total peak area of that sample and then multiplied by the mean of the total peak areas across all samples. To account for potential batch effects, if samples are analyzed in multiple batches, QC-based batch correction was performed using the median values across batches. Instrument stability was assessed using the relative standard deviation (RSD) of metabolite peak areas in the QC samples; metabolites with an RSD > 30% in the QC samples were excluded.

According to the criteria of VIP > 1.0, Fold Change > 1.5 or Fold Change < 0.667 and P-value < 0.05, differential metabolites were screened (Sreekumar et al., 2009; Haspel et al., 2014; Heischmann et al., 2016).

### 2.10 Data analysis

All samples contained three biological replicates. First, a two-way analysis of variance (ANOVA) was used to examine the effects of storage temperature, storage time, and their interaction (temperature × time) on each parameter. When the interaction was significant, a simple effects analysis was conducted. Differences between the means of different treatments were analyzed using Duncan’s method, and the data were expressed as the mean ± standard error. Significant differences between the groups were determined at *P* < 0.05.

## 3 Results

The results of the two-way ANOVA indicated that the interaction between storage temperature and storage duration had a significant effect (*P* < 0.05) on the rot rate, relative electrical conductivity, respiration rate, MDA content, SDH activity, and CAT activity of the truffles, suggesting that the effect of temperature on truffle quality indicators is not constant across different storage stages but varies with storage duration. Therefore, a one-way ANOVA was conducted with storage time held constant to identify the specific differences among different temperature treatments at the same storage time (**Supplementary Tables 2**–**7**).

### 3.1 Effects of different storage temperatures on decay rate

**Figure 1:**
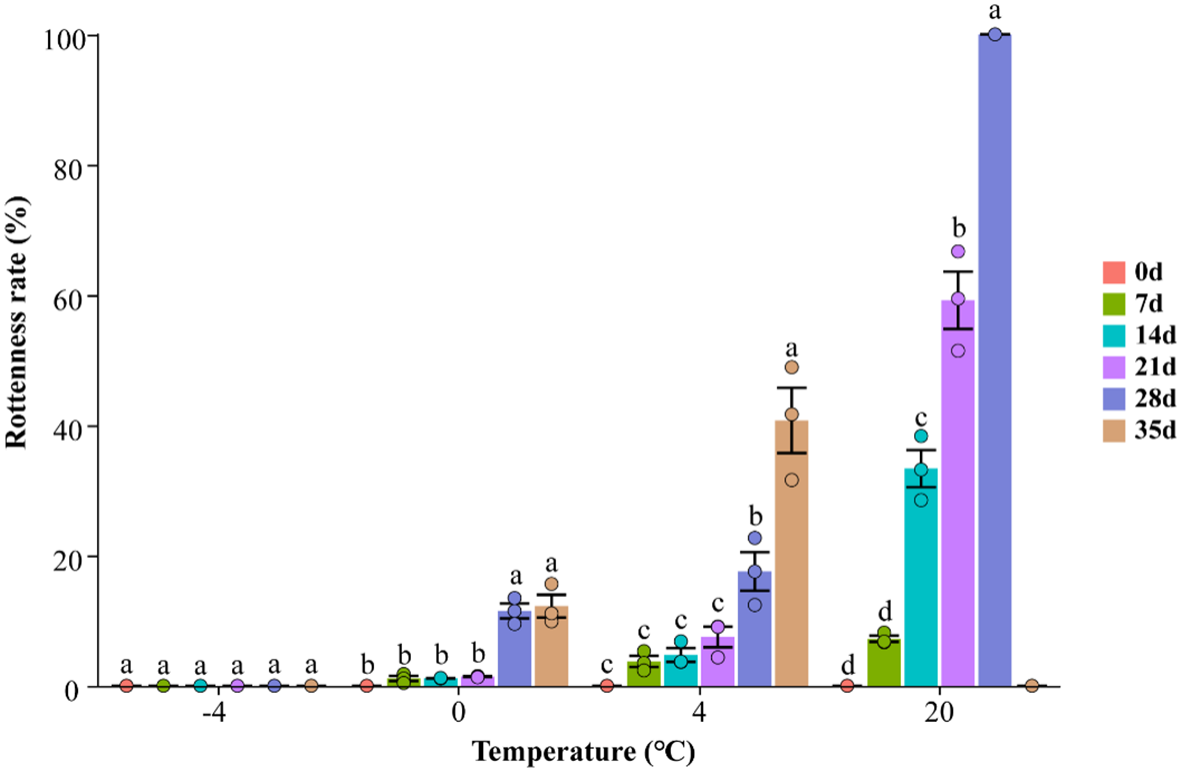
Rottenness rate of samples in different storage time groups. There were significant differences between groups for the same storage temperature but different storage durations, as indicated by different letters (*P* < 0.05).

Rotting is a common cause of quality changes in edible fungi during storage, mainly manifested as mold growth, stickiness, and odor on the surface of truffles. As the storage period extended to day 35, no rotting occurred in truffles stored at -4 °C, indicating good preservation effects (**Figure 1**). However, under 0 °C, 4 °C, and 20 °C storage conditions, rotting appeared in truffles by day 7, with rotting rates of 1.11 ± 0.39%, 3.72 ± 0.86%, and 7.20 ± 0.47%, respectively (*P* < 0.05). For truffles stored at 0 °C, the rotting rate reached 11.48 ± 1.15% by day 28, showing a significant increase compared to days 0, 7, 14, and 21 (*P* < 0.05). Under 4 °C storage, the rotting rate of truffles significantly increased to 7.49 ± 1.57% by day 21 (*P* < 0.05). For truffles stored at 20 °C, the rotting rate reached 100% by day 28, marking a significant rise compared to earlier time points (*P* < 0.05). The experimental results demonstrate that low-temperature storage effectively reduces the rotting rate of truffles during storage, slows the decline in quality, and maintains the quality and flavor of truffles.

### 3.2 Effects of different storage temperatures on relative conductivity

**Figure 2:**
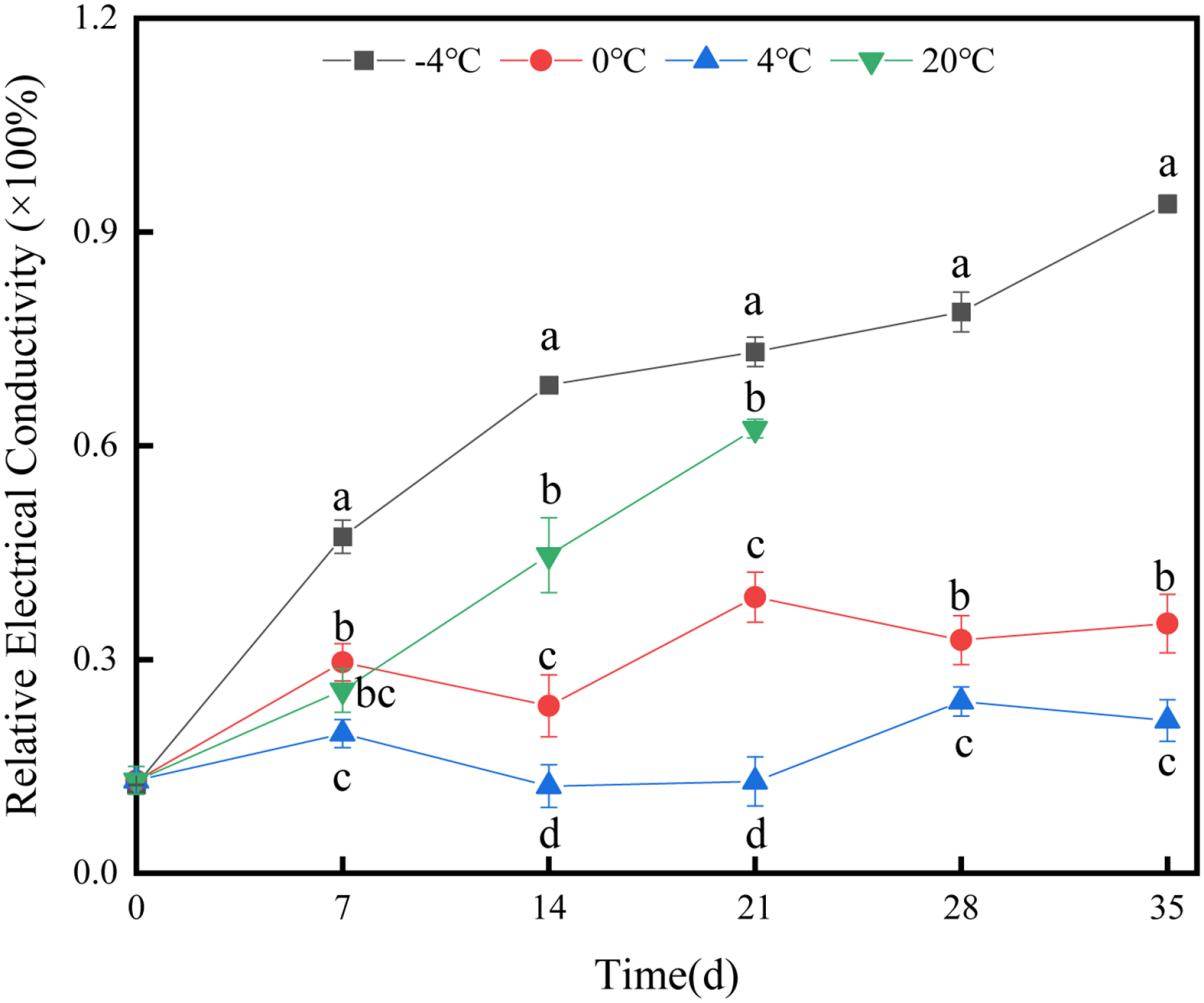
Changes in the relative electrical conductivity of truffles stored at different temperatures. The different letters indicate that, for the same storage duration, there are significant differences between groups at different storage temperatures. (*P* < 0.05).

When stored at -4 °C, the relative conductivity of truffles showed an overall upward trend from day 0 to day 21, and the increase leveled off from day 21 to day 35. Overall, the relative conductivity of truffles stored at -4 °C remained consistently higher than that of truffles stored at other temperatures (**Figure 2**); when stored at 20 °C, the relative conductivity rose sharply. On day 7, truffles stored at 4 °C exhibited the lowest relative conductivity, at 0.20 ± 0.02% (*P* < 0.05). When the storage period was extended to day 14, truffles stored at –4 °C had the highest relative conductivity, at 0.47 ± 0.02% (*P* < 0.05). When the storage period was extended to day 35, the relative conductivity of truffles stored at −4 °C was the highest, at 0.94 ± 0.01% (*P* < 0.05). Furthermore, for truffles stored at 0 °C and 4 °C, the increase in relative conductivity was relatively stable over time; during the same period, their relative conductivity was lower compared to the other two groups.

### 3.3 Effects of different storage temperatures on respiration rate

**Figure 3:**
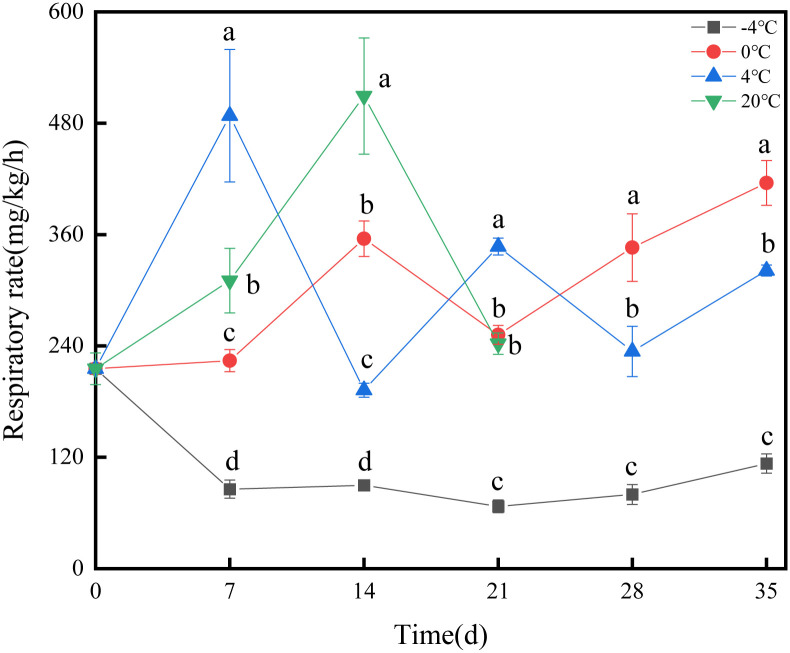
Effects of different storage temperatures on respiratory rates. The different letters indicate that, for the same storage duration, there are significant differences between groups at different storage temperatures. (*P* < 0.05).

Under conditions of -4 °C, the respiratory rate of the truffles was relatively low and increased gradually. On day 7, the highest respiration rate occurred in the -4 °C group, at 224.04 ± 11.89 mg/kg/h (*P* < 0.05) (**Figure 3**). As the storage period extended to 14 days, the respiration rate of truffles stored at 20 °C was the highest, at 509.29 ± 62.60 mg/kg/h (*P* < 0.05). (**Figure 3**). As the storage period extended to 21 days, the respiratory rate of truffles in the –4 °C group was the highest, at 347.09 ± 9.09 mg/kg/h (*P* < 0.05). By day 35 of storage, the respiratory rate of truffles stored at 0 °C was the highest (*P* < 0.05).

### 3.4 Changes in the microstructure of truffle tissues under different storage temperatures

**Figure 4:**
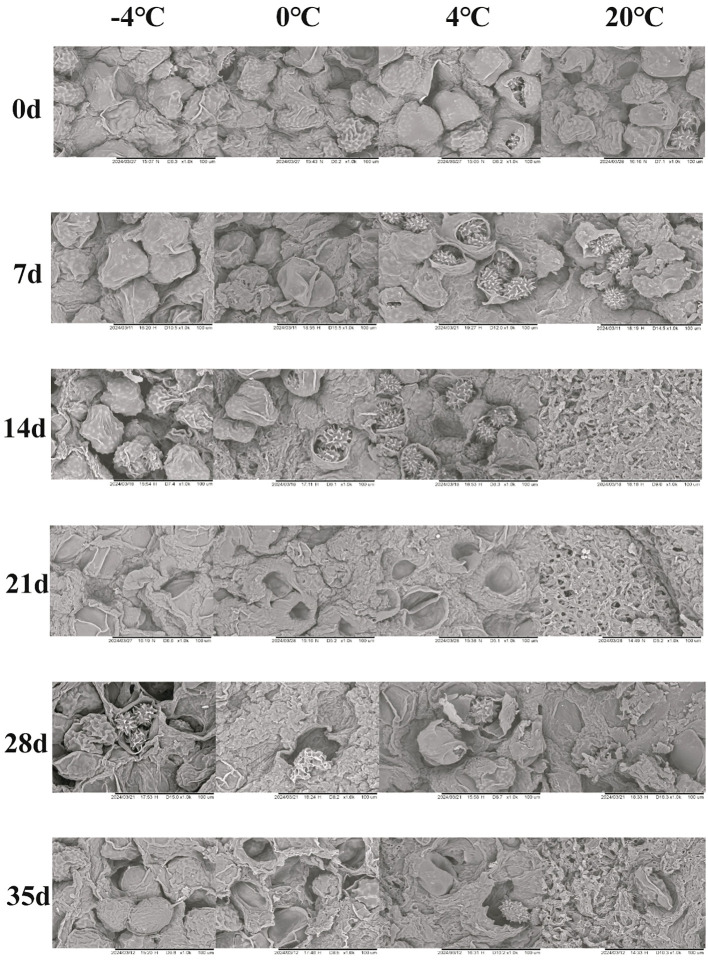
Scanning electron microscope images of truffle slices under different storage conditions.

To further investigate the changes in its microstructure, scanning electron microscopy was used to observe the changes in truffles (**Figure 4**). On the 0th day, the truffles had a uniform texture, with intact and plump sporangia and minimal rupture. When stored until 7 d, the sporangia of truffles stored at -4 °C remained intact, while those stored at 0 °C showed localized collapse or breakage of the cell walls, and dissolution of the matrix between hyphae. The sporangia of truffles stored at 4 °C exhibited relatively severe damage, followed by those stored at 20 °C. By 14 d, the sporangia of truffles stored at -4 °C still maintained a relatively intact state. Those stored at 0 °C showed significant damage, but the degree of degradation was somewhat alleviated. Truffles stored at 20 °C displayed obvious dissolution of the ascus walls and numerous pores. When stored until 21 d, the degradation of ascus walls in truffles stored at 0 °C worsened again. The structure of truffles stored at 4 °C was also severely damaged, with spore walls shedding and visible large pore depressions, and the largest pores. Truffles stored at 20 °C exhibited a multi-layered stacked network structure. By 35 d, the ascus walls of truffles stored at 4 °C showed the most severe degradation, with severely fragmented structures and large pores. The ascus walls of truffles stored at -4 °C had just begun to show damage. Those stored at 0 °C had severely damaged surfaces with noticeable pits.

### 3.5 Effects of different storage temperatures on MDA content

**Figure 5:**
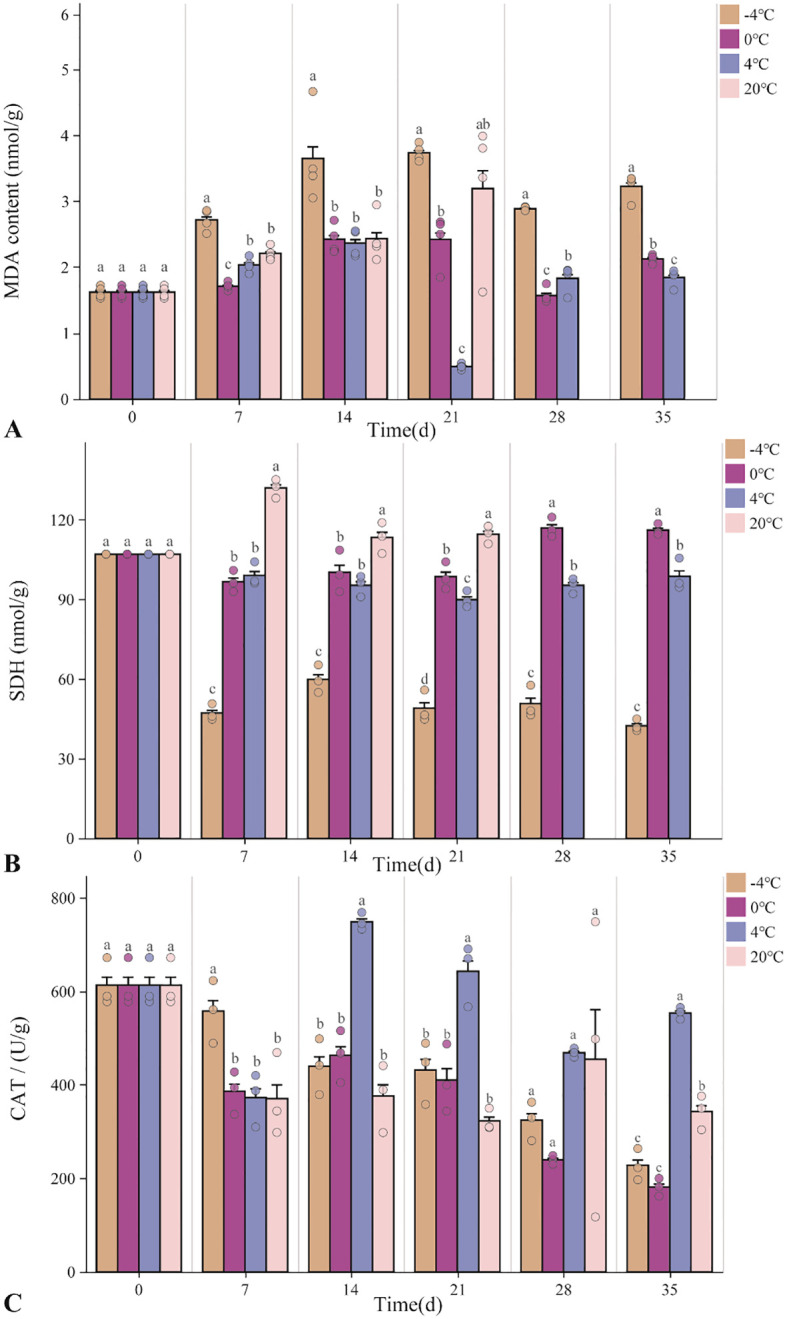
Changes in truffles enzyme activity at different storage temperatures. **(A)** Changes in MDA content in truffles at different storage temperatures. **(B)** Changes in SDH content in truffles at different storage temperatures. **(C)** Changes in CAT content in truffles at different storage temperatures (*P* < 0.05).

MDA is the main product of membrane lipid peroxidation, which can affect the structure of truffle membranes and disrupt normal physiological metabolism. It is commonly used to assess the degree of cell membrane damage and is directly proportional to the level of cellular oxidative damage. Changes in MDA levels can serve as an indicator of plant cold tolerance. Generally, an increase in MDA content reduces plant cold tolerance. The study found (**Figure 5A**) that, except for no significant difference in MDA content of truffles stored at various temperatures on 0 d (*P*>0.05). The MDA content of truffles stored at -4 °C was significantly higher than those stored at other temperatures during other storage periods(*P* < 0.05). On 7 d, the MDA content of truffles stored at 0 °C was the lowest, at 1.71 ± 0.06 nmol/g (*P* < 0.05). On 21 d, the MDA content of truffles stored at 4 °C was the lowest, at 0.49 ± 0.04 nmol/g (*P* < 0.05). On 28 d, the MDA content of truffles stored at -4 °C was the highest, at 2.89 ± 0.03 nmol/g (*P* < 0.05). On 35 d, the MDA content of truffles stored at -4 °C was the highest, at 3.23 ± 0.20 nmol/g (*P* < 0.05).

### 3.6 Effects of different storage temperatures on SDH activity

Except for 0 d, the SDH activity of truffles stored at -4 °C showed no significant difference compared to those stored under other temperature conditions (*P*>0.05) (**Figure 5B**). However, at other time points, the SDH activity of truffles stored at -4 °C was significantly lower than that of the room temperature group (*P* < 0.05). As the storage time increased to the 7th day, the study found that the SDH activity of truffles stored at 20 °C was the highest, reaching 132.05 ± 3.52 U/g (*P* < 0.05). Additionally, at 14 d and 21 d, the SDH activity of truffles stored at 20 °C was significantly higher than other groups, with values of 113.38 ± 5.84 U/g and 114.61 ± 3.36 U/g, respectively (*P* < 0.05). By the 28th and 35th day, the study found that the SDH activity of truffles stored at 0 °C was the highest, at 114.97 ± 3.71 U/g and 116.99 ± 2.15 U/g, respectively(*P* < 0.05). Overall, as the storage time extended, the SDH activity of truffles stored at -4 °C and 4 °C showed a downward trend, while those stored at 0 °C and 20 °C exhibited an upward trend.

### 3.7 Effects of different storage temperatures on CAT activity

Over time, the CAT activity of all sample groups showed a declining trend (**Figure 5C**). On the 7th day, the CAT content of truffles stored at -4 °C was 624.63 ± 67.10 U/g, significantly higher than the other groups (*P* < 0.05). On 14th, 21th, a360hangnd 35th day, the CAT content of truffles stored at 4 °C was significantly higher than other groups (*P* < 0.05). On 28th day, there was no significant difference in CAT content among truffles stored at different temperatures (*P* > 0.05).

### 3.8 Metabolomics analysis

#### 3.8.1 Metabolite level 1 classification and PCA analysis

In the primary metabolites under positive ion mode (**Supplementary Figure 1A**), there are 212 types of lipids and lipid-like molecules (29.44%), 174 types of organic acids and derivatives (24.17%), 112 types of organoheterocyclic compounds (15.56%), 67 types of benzenoids (9.31%), 44 types of nucleosides, nucleotides, and analogues (6.11%), 37 types of organic oxygen compounds (5.14%), 31 types of phenylpropanoids and polyketides (4.31%), 23 types of organic nitrogen compounds (3.19%), 12 types of alkaloids and derivatives (1.67%), 2 types of organosulfur compounds (0.28%), and 1 type of hydrocarbons (0.14%).In the primary metabolites under negative ion mode (**Supplementary Figure 1B**), there are 183 types of lipids and lipid-like molecules (36.75%), 115 types of organic acids and derivatives (23.09%), 56 types of organic oxygen compounds (11.24%), 52 types of nucleosides, nucleotides, and analogues (10.44%), 40 types of organoheterocyclic compounds (8.03%), 27 types of benzenoids (5.42%), 18 types of phenylpropanoids and polyketides (3.61%), 5 types of organic nitrogen compounds (1.00%), and 1 type of alkaloids and derivatives (0.20%).

PCA, as an unsupervised pattern recognition method, is commonly used to reflect the similarity of sample metabolites and identify analytical methods for abnormal differences. The more concentrated the sample groups are, the smaller the differences. In positive ion mode, the 4 °C vs 0 °C group accounted for 84% and 6% of the sample variance in PC1 and PC2, respectively (**Supplementary Figure 2A**); the -4 °C vs 0 °C samples accounted for 84% and 8% of the sample variance in PC1 and PC2, respectively (**Supplementary Figure 2B**); and the -4 °C vs 4 °C samples accounted for 79% and 8% of the sample variance in PC1 and PC2, respectively (**Supplementary Figure 2C**). There was no overlap between the groups, significant separation, and significant differences between each pair. In negative ion mode, the 4 °C vs 0 °C group accounted for 80% and 11% of the sample variance in PC1 and PC2, respectively (**Supplementary Figure 3A**). The -4 °C vs 0 °C samples accounted for 82% and 9% of the sample variance in PC1 and PC2, respectively. (**Supplementary Figure 3B**). The -4 °C vs 4 °C samples accounted for 81% and 8% of the sample variance in PC1 and PC2, respectively (**Supplementary Figure 3C**). This indicates that the metabolites in all samples underwent a certain degree of separation.

#### 3.8.2 Differential metabolite analysis

**Figure 6:**
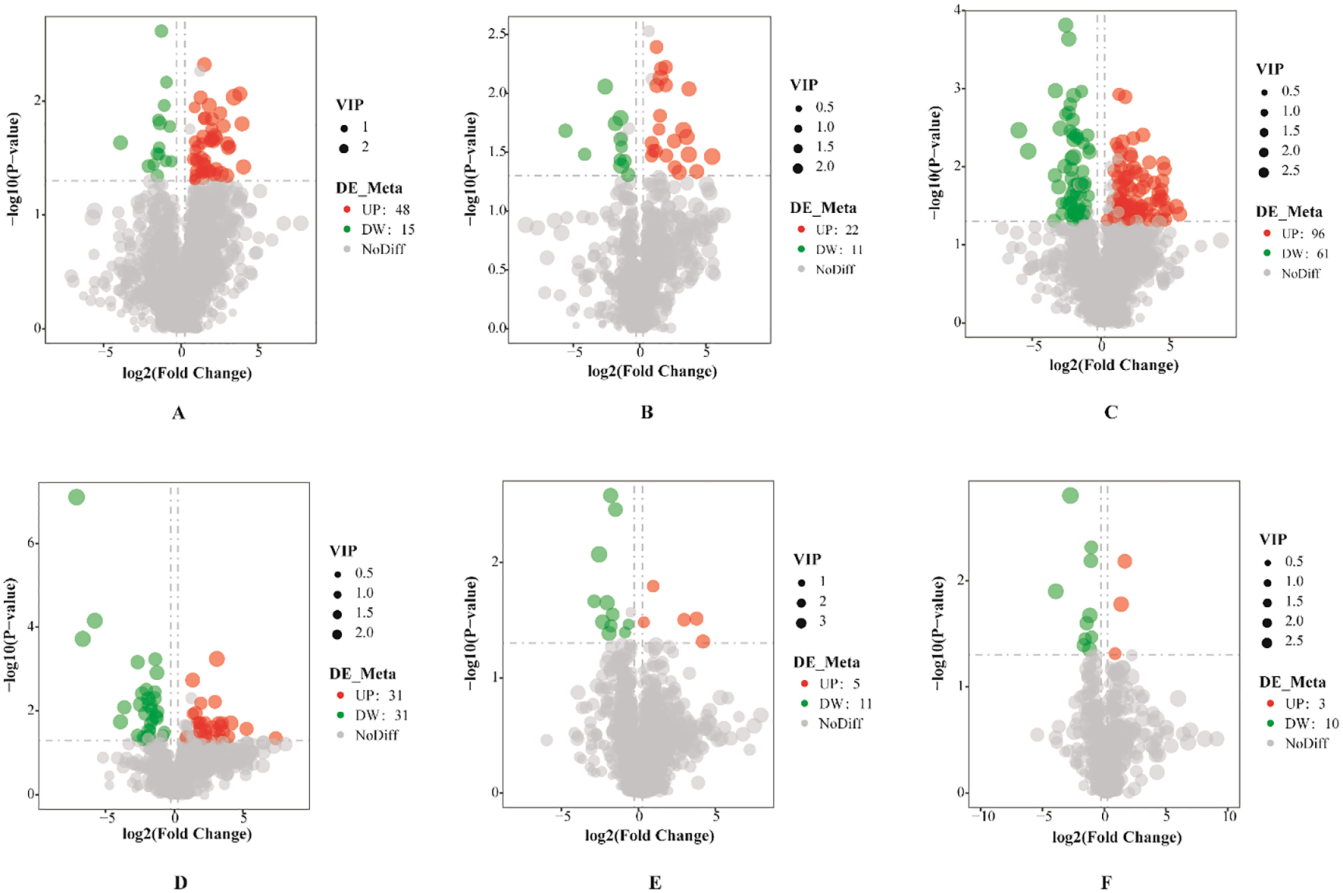
Differences in metabolite expression in truffles at different temperatures. **(A)** 4 °C vs 0 °C group bubble chart in positive ion mode **(B)** 4 °C vs 0 °C group bubble chart in negative ion mode **(C)** 4 °C vs -4 °C group bubble chart in positive ion mode **(D)** 4 °C vs -4 °C group bubble chart in negative ion mode **(E)** 0 °C vs -4 °C group bubble chart in positive ion mode **(F)** 0 °C vs -4 °C group bubble chart in negative ion mode.

Compared to the 0 °C group, 63 DEMs were identified in the positive ion mode in the 4 °C group, with 48 DEMs significantly upregulated and 15 DEMs significantly downregulated (**Figure 6A**);In the negative ion mode, 33 DEMs were found, with 22 DEMs significantly upregulated and 11 DEMs significantly downregulated (**Figure 6B**).Compared to the 4 °C group, 157 DEMs were detected in the positive ion mode in the -4 °C group, with 96 DEMs significantly upregulated and 61 DEMs significantly downregulated (**Figure 6C**). In the negative ion mode, 62 DEMs were identified, with 31 DEMs significantly upregulated and 31 DEMs significantly downregulated (**Figure 6D**).Compared to the 0 °C group, 16 DEMs were observed in the positive ion mode in the -4 °C group, with 5 DEMs significantly upregulated and 11 DEMs significantly downregulated (**Figure 6E**). In the negative ion mode, 13 DEMs were found, with 3 DEMs significantly upregulated and 10 DEMs significantly downregulated (**Figure 6F**). Subsequent research focused on the five metabolites with the highest VIP values and smallest P-values in each group. In the 4 °C vs. 0 °C comparison, the most significant metabolites in the positive ion mode were 2-Methylpentanedioic acid, Furanyl fentanyl 3-furancarboxamide isomer-d5, N-Acetyl-D-tryptophan, Ergocalciferol, and N-Formylkynurenine (*P* < 0.05). In the negative ion mode, the most significant metabolites were Taurine, Dithranol, 2-(2-oxo-2-{[2-(2-oxo-1-imidazolidinyl)ethyl]amino}ethoxy)acetic acid, 2-Isopropylmalic acid, and 3-Isopropylmalic acid (*P* < 0.05). In the -4 °C vs. 4 °C comparison, the most significant metabolites in the positive ion mode were 1-[2-(2,5-dimethyl-1H-pyrrol-1-yl)-4-nitrophenyl]-1H-imidazole, 2-Aminopimelic acid, 6-{[2-(2-oxo-1-imidazolidinyl)ethyl]amino}-2-pyridinecarbonitrile, γ-Aminobutyric acid (GABA), and D-(+)-Tryptophan (*P* < 0.05). In the negative ion mode, the most significant metabolites were 4-Methylvaleric Acid, Equol, N,N-dimethyl-N’-(2-thienylmethyl)thiourea, Dithranol, and 2-C-methyl D-erythritol 4-phosphate (P <0.05). In the -4 °C vs. 0 °C comparison, the most significant metabolites in the positive ion mode were 3-(4-nitrophenyl) (Wang and Marcone, 2011; Herrero de Aza et al., 2022; Angeloni et al., 2025) triazolo[1,5-a]quinazolin-5-amine, methyl 1-methyl-1,2,5,6-tetrahydropyridine-3-carboxylate hydrobromide, 1-[2-(2,5-dimethyl-1H-pyrrol-1-yl)-4-nitrophenyl]-1H-imidazole, VMK, and [5-(2-thienyl)-3-isoxazolyl]methanol (*P* < 0.05).In the negative ion mode, the most significant were N-Acetyl-L-glutamine, N-Sulfo-glucosamine sodium salt, Theophylline, Glycerol 3-phosphate, and glutathione disulfide (*P* < 0.05).

#### 3.8.3 KEGG functional enrichment analysis

**Figure 7:**
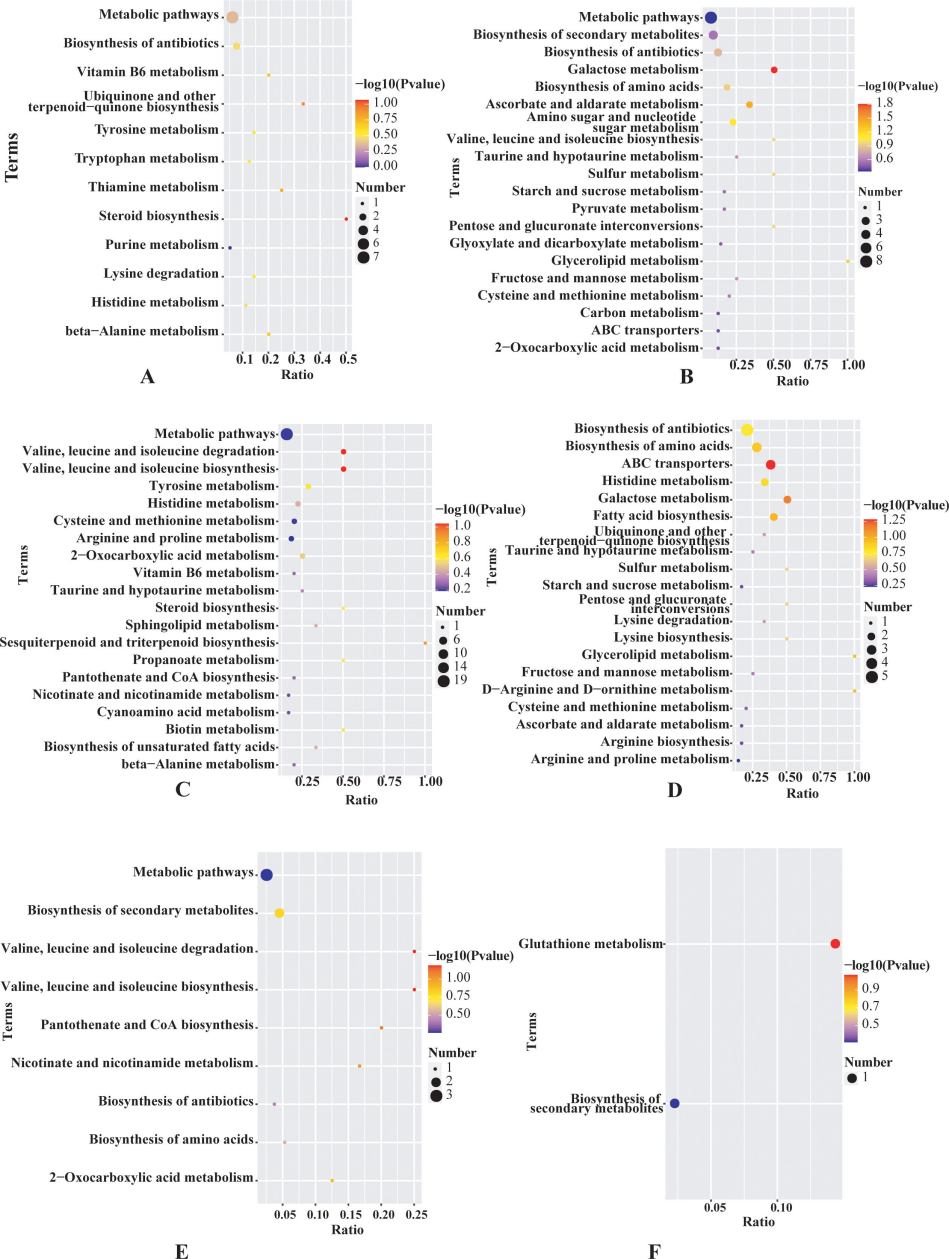
KEGG enrichment bubble chart of metabolic differences in truffles at different temperatures. **(A)** 4 °C VS 0 °C group bubble chart in positive ion mode **(B)** 4 °C VS 0 °C group bubble chart in negative ion mode **(C)** 4 °C VS -4 °C group bubble chart in positive ion mode **(D)** 4 °C VS -4 °C group bubble chart in negative ion mode **(E)** 0 °C VS -4 °C group bubble chart in positive ion mode **(F)** 0 °C VS -4 °C group bubble chart in negative ion mode.

Functional annotation and enrichment analysis was performed on the screened differential metabolites. The main pathways involved in the differential metabolites of each comparison group were represented by bubble plots. In the 4 °C vs 0 °C group comparison, the positive ion mode showed significant enrichment in steroid biosynthesis, ubiquinone and other terpenoid-quinone biosynthesis, thiamine metabolism, beta-alanine metabolism, and vitamin B6 metabolism (*P* < 0.05) (**Figure 7A**). Negative ion mode revealed significant enrichment in galactose metabolism, ascorbate and aldarate metabolism, glycerolipid metabolism, amino sugar and nucleotide sugar metabolism, and pentose and glucuronate interconversions (*P* < 0.05) (**Figure 7B**). In the -4 °C vs 4 °C group comparison, the positive ion mode primarily exhibited enrichment in valine, leucine, and isoleucine degradation, valine, leucine, and isoleucine biosynthesis, sesquiterpenoid and triterpenoid biosynthesis, tyrosine metabolism, and steroid biosynthesis (*P* < 0.05) (**Figure 7C**). While negative ion mode showed enrichment in ABC transporters, galactose metabolism, fatty acid biosynthesis, D-arginine and D-ornithine metabolism, and glycerolipid metabolism (*P* < 0.05) (**Figure 7D**). In the -4 °C vs 0 °C group comparison, the positive ion mode demonstrated enrichment in valine, leucine, and isoleucine degradation, valine, leucine, and isoleucine biosynthesis, pantothenate and CoA biosynthesis, nicotinate and nicotinamide metabolism, and 2-oxocarboxylic acid metabolism (*P* < 0.05) (**Figure 7E**), whereas the negative ion mode indicated enrichment in glutathione metabolism and biosynthesis of secondary metabolites (*P* < 0.05) (**Figure 7F**).

## 4 Discussion

### 4.1 Effects of low-temperature storage on the decay rate and cell membrane integrity of truffles

Truffles stored at -4 °C showed no decay within 35 days, while those stored at 20 °C (room temperature) reached a 100% decay rate by 28 days. This aligns with the notion that low temperatures delay postharvest spoilage by inhibiting microbial proliferation and reducing metabolic activity (Sheng et al., 2021; Kaur et al., 2022). Kong et al. (2025) found in their study on *Phlebopus portentosus* that the postharvest quality deterioration rate under 4 °C storage was significantly lower than under 25 °C (room temperature) storage, further confirming that low temperatures can synergistically suppress metabolic activity and microbial growth, thereby maintaining the postharvest quality of edible fungi. However, in this study, the relative electrical conductivity of truffles in the -4 °C treatment group remained significantly higher than in other temperature groups throughout the storage period, rising to 1.01 ± 0.06% by 21th day. Although near-freezing storage effectively delays the senescence of fruiting bodies, it exacerbates intracellular electrolyte leakage and increases cell membrane permeability (Zheng et al., 2022). Relative electrical conductivity is a key indicator for assessing the integrity of the cell membrane system (Bajji et al., 2002). An increase in this value suggests a phase transition in the cell membrane from a liquid crystalline state to a gel state, accompanied by a decrease in the unsaturation of membrane lipid fatty acids, ultimately leading to structural membrane damage (Sati et al., 2025). Truffles stored at -4 °C may face cold injury risks; while low temperatures effectively inhibit postharvest decay, inappropriate low-temperature stress can induce membrane phase transitions, causing abnormal increases in membrane permeability.

### 4.2 Effects of low temperature on respiration rate and energy metabolism of truffles

Under -4 °C storage conditions, the respiration rate of truffles remained at the lowest level throughout, and increased slowly with prolonged storage time. This aligns with the core mechanism in which low temperature reduces the kinetic energy of enzymes and slows the diffusion rates of substrates, thereby inhibiting respiratory metabolism. Yang et al. (2026) demonstrated in their study on *Volvariella volvacea* that the respiratory intensity of its fruiting bodies under 15 °C storage was significantly lower than that at room temperature. Additionally, pre-cooling with cold air could downregulate the activities of key tricarboxylic acid (TCA) cycle enzymes such as succinate dehydrogenase (SDH), isocitrate dehydrogenase, and citrate synthase, thereby limiting TCA cycle flux and maintaining energy metabolic homeostasis in fungal bodies. In this study, the SDH activity of the -4 °C treatment group continuously decreased with prolonged storage time, dropping to 25.65 U·g^−1^ by day 35 of storage. This result is consistent with the aforementioned findings (Yang et al., 2026), further confirming that low temperature can suppress the activity of key TCA cycle enzymes, reduce the energy metabolism level of fungal bodies, and thereby delay postharvest senescence processes.

However, the respiration rates of truffles in the 0 °C and 4 °C treatment groups exhibited abnormal fluctuations with prolonged storage time. Notably, the 4 °C treatment group showed a significant respiratory peak on the 7th day of storage, reaching a maximum value of 488.08 μmol·g^−1^·h^−1^. This phenomenon is consistent with the findings of Wang et al (Sun et al., 2024). where the respiration rate of postharvest edible fungi initially decreased, then increased, and subsequently decreased over storage time. However, ϵ-polylysine treatment significantly inhibited the occurrence of respiratory peaks. The abnormal fluctuations in respiration rate may be related to the activation of non-phosphorylated respiratory bypass pathways, such as the alternative oxidase (AOX) pathway, in truffle cells under low-temperature stress. When the classical mitochondrial electron transport chain is blocked, the AOX-mediated alternative respiratory pathway is activated to maintain intracellular energy supply homeostasis, leading to an increase in respiration rate (Sun et al., 2024). Additionally, the SDH activity of truffles in the 0 °C treatment group showed an upward trend with prolonged storage time and was significantly higher than that of the -4 °C treatment group in the later storage period. This suggests a more vigorous energy metabolism in the fungal bodies under this temperature condition, thereby accelerating the maturation of fruiting bodies and the postharvest senescence process. Research has found that storage at -4 °C minimizes spoilage while simultaneously resulting in the highest levels of relative electrical conductivity and malondialdehyde (MDA) accumulation. This phenomenon may seem contradictory, but it actually reveals the trade-off between “preservation” and “freeze damage prevention” in the low-temperature storage of truffles. Relative electrical conductivity and malondialdehyde (MDA) levels reflect physical and oxidative damage to cell membranes, while “decay” primarily results from microbial activity and autolysis; at -4 °C, the former is most severe, while the latter is most effectively suppressed.1. At -4 °C, cell membrane fluidity is extremely low, metabolic activity virtually ceases, and microorganisms are unable to reproduce or secrete degradative enzymes; consequently, decay is naturally suppressed to the greatest extent. Since 4 °C is below the tissue freezing point, the activity of most enzymes (such as polyphenol oxidase and proteases) is strongly inhibited, thereby reducing spoilage.2. Increased relative conductivity → Severe damage to cell membranes. Truffle fruiting bodies have a very high water content (often exceeding 80%), and their tissue freezing point is generally between -1 and -2 °C. Storage at -4 °C causes truffles to freeze, resulting in the formation of ice crystals both inside and outside the cells. Ice crystals mechanically puncture cell membranes; upon thawing, membrane integrity is lost, causing massive efflux of intracellular electrolytes and leading to a sharp rise in the conductivity of the soaking solution. The lower the temperature, the more severe the ice crystal damage, and consequently, the higher the relative conductivity.3. Peak MDA Accumulation → Exacerbated Membrane Lipid Peroxidation. The freeze-thaw process and prolonged low-temperature stress induce the massive production of reactive oxygen species (ROS). At the same time, the activity of antioxidant enzymes (such as SOD and CAT) is inhibited at low temperatures. When ROS cannot be scavenged, they attack the unsaturated fatty acids in cell membranes, triggering lipid peroxidation and leading to a massive accumulation of the end product, MDA. When the temperature drops to -4 °C, this oxidative damage is typically most severe, resulting in the highest MDA levels.

### 4.3 Effects of low temperature on oxidative damage and antioxidant system in truffles

Malondialdehyde (MDA) is the end product of membrane lipid peroxidation, and its content reflects the degree of oxidative damage to the cell membrane system (Liu et al., 2021). In this study, the MDA content of truffles in the -4 °C treatment group was significantly higher than that in the other low-temperature treatment groups during the middle and late stages of storage. During low-temperature storage, the MDA content and electrolyte leakage rate showed a synchronized slow upward trend (Zheng et al., 2022), both indicating a continuous intensification of cell membrane lipid peroxidation. However, the overall decay rate of truffles in the -4 °C treatment group was 0, suggesting that low temperatures can inhibit microbial infection (Silva et al., 2025), proliferation, and enzymatic browning, thereby maintaining the postharvest spoilage process and keeping the truffle fruiting bodies in a relatively stable state. This phenomenon aligns with the general principles of postharvest physiology in edible fungi: although low temperatures can inhibit basal metabolic processes by reducing enzyme activity (Wu et al., 2024), they must be combined with exogenous antioxidants and other agents to scavenge excess reactive oxygen species within cells in order to effectively alleviate cold-induced oxidative damage.

Catalase (CAT) is a core antioxidant enzyme responsible for intracellular clearance of H_2_O_2_, and changes in its activity can reflect the functional state of the antioxidant defense system in bacterial cells (Rajput et al., 2021). In this study, the CAT activity of truffles in all treatment groups showed a continuous downward trend with prolonged storage time, consistent with the pattern of gradual decline in the antioxidant system function of postharvest edible fungi over storage duration (Yang et al., 2025). Notably, the CAT activity of the -4 °C treatment group remained significantly higher than that of the other treatment groups during the first 21 days of storage, suggesting that truffles could activate their antioxidant defense system to cope with low-temperature stress in the early storage phase. However, by day 35 of storage, their CAT activity dropped sharply and became significantly lower than that of the 4 °C treatment group. Previous studies have found that SOD activity in samples stored at 10 °C is higher than in those stored at 5 °C (Dama et al., 2010). This is because oxidative stress increases when samples are stored at higher temperatures, thereby inducing the production of antioxidant enzymes (Karimirad et al., 2020). Near-freezing storage during the initial phase can induce an increase in antioxidant enzyme activity (Zheng et al., 2022), alleviating oxidative stress caused by reactive oxygen species. However, prolonged low-temperature stress can deplete the antioxidant defense capacity, accelerating the senescence of fruiting bodies. The CAT activity of the 4 °C treatment group was significantly higher than that of the other groups during the middle and late storage periods (14th, 21th, and 35th days), indicating that this storage temperature offers greater advantages in maintaining long-term antioxidant defense capacity in truffles. This may be related to the milder cold stress and relatively stable maintenance of antioxidant enzyme structure and function at this temperature (Dong et al., 2025).

### 4.4 Differences in the microstructure of truffles under different storage temperatures

Scanning electron microscopy (SEM) observation results showed that when stored for 35th day, the ascocarps of the -4 °C treatment group exhibited only minor damage, and the overall microstructure of the fruiting bodies remained intact. This aligns with the mechanism by which low temperatures maintain cellular structural integrity by reducing respiratory metabolic rates and inhibiting cell wall-degrading enzyme activity (Wang et al., 2026). In contrast, the ascocarps of the 4 °C treatment group showed the most severe degradation of the ascus walls and significant pore formation in the structure after 35th day of storage. This is similar to previous findings (Zheng et al., 2022), where low-temperature storage could maintain basic cellular morphology, but the activities of cell wall-modifying enzymes such as cellulase and chitinase differed significantly under different storage temperatures, ultimately leading to varying degrees of cellular structural damage. The study by Savini et al. (2020) also confirmed that low-temperature storage significantly affects the antioxidant properties and volatile substance metabolism of truffles, with a remodeling of membrane lipid composition being the core mechanism for truffles to cope with cold stress and achieve cold adaptation.

Under 20 °C ambient storage conditions, the microstructure of truffle fruiting bodies exhibited a multi-layered, stacked network morphology, which may result from the combined effects of microbial invasion and autolysis of fungal tissues under high-temperature conditions. The study by Kong et al. (2025) has confirmed that during room-temperature storage, microbial proliferation in *Phlebopus portentosus* is vigorous, and the cell wall-degrading enzymes they secrete severely damage the tissue structure of the fruiting bodies, ultimately leading to surface stickiness and off-odor production. This is highly consistent with the observations in this study.

### 4.5 Metabolic reprogramming mechanisms of truffles under low-temperature stress

The results of non-targeted metabolomics analysis showed that the -4 °C vs 4 °C comparison group had the highest number of differential metabolites (DEMs), with 157 DEMs identified in positive ion mode. This indicates that the greater the temperature span during storage, the more significant the perturbation effect on the truffle metabolome. The branched-chain amino acid (BCAA) metabolic pathway was significantly enriched in both the -4 °C vs 0 °C and -4 °C vs 4 °C comparison groups. Zhao et al (Tejedor-Calvo et al., 2024). elucidated the response mechanism of straw mushrooms to low-temperature stress through metabolomics, finding that cold-tolerant strains enhance their cold resistance by regulating amino acid metabolism and energy metabolic pathways. BCAAs are not only core precursors for protein biosynthesis, but they also play a significant role in intracellular energy metabolism and oxidative stress responses. The significant changes in their metabolic levels reflect that truffles can maintain intracellular energy supply and redox homeostasis under low-temperature stress by remodeling the amino acid metabolic network.

In negative ion mode, the glutathione metabolic pathway was significantly enriched in the -4 °C vs 0 °C comparison group. Glutathione (GSH) is the most important non-enzymatic antioxidant substance in cells (Forman et al., 2009; Li et al., 2022). The significant enrichment of this pathway suggests that under -4 °C storage conditions, truffles are subjected to strong oxidative stress, requiring the upregulation of GSH synthesis metabolism to clear excessive intracellular reactive oxygen species (ROS) (Savini et al., 2020). This result aligns with the earlier observation of a significant increase in CAT activity during the initial storage period, followed by a rapid decline in the later stage. Both confirm that prolonged low-temperature stress leads to the gradual exhaustion of the antioxidant defense system in truffles. Zhou et al. (2025) found that CaCl_2_ treatment can enhance the intracellular ROS scavenging capacity by activating key genes in the ascorbate-glutathione cycle, thereby alleviating postharvest chilling injury in fruits and vegetables. This suggests that appropriate exogenous substance treatments may mitigate oxidative stress and chilling injury in truffles stored at -4 °C.

In positive ion mode, the steroid biosynthesis pathway was significantly enriched in the -4 °C vs 4 °C comparison group. Steroids are closely related to the regulation of cell membrane fluidity, and their significant changes in synthesis and metabolism may be an important adaptive response of truffles to low-temperature-induced cell membrane phase transitions. Savini et al. (2020) also found that low-temperature storage can significantly affect the membrane lipid composition and antioxidant properties of truffles, and membrane lipid remodeling is a core mechanism for maintaining cell membrane fluidity under low-temperature conditions. In addition, the ABC transporter pathway was also significantly enriched in the -4 °C vs. 4 °C comparison group in negative ion mode; this may lead to changes in the transport function of truffle cell membranes under -4 °C storage conditions, and may also be related to the fungus’s ability to respond to membrane damage caused by low temperatures and maintain the homeostasis of the intracellular microenvironment (Sati et al., 2025).

## 5 Conclusion

This study focuses on Sichuan Huidong truffles as the research subject, investigating the changes in postharvest decay rate, relative electrical conductivity, and respiratory rate under different storage temperatures. Metabolomic analysis was conducted to screen for differential metabolites and enrich related metabolic pathways. Research has found that storage at -4 °C completely inhibits tissue decay on the surface of truffles, with a decay rate of 0% over a 35-day storage period; therefore, this temperature is more suitable for storage. The relative electrical conductivity and malondialdehyde (MDA) content of the -4 °C treatment group were significantly higher than those of the 0 °C and 4 °C treatment groups throughout the storage period (*P* < 0.05). The respiratory rate of truffles under -4 °C conditions was relatively low and increased gradually. Scanning electron microscopy revealed that the ascus walls of truffles stored at -4 °C began to show slight damage on 35th day. Metabolomic analysis identified significant enrichment of the steroid biosynthesis pathway in both the 4 °C vs 0 °C and -4 °C vs 4 °C groups under positive ion mode. Additionally, the valine, leucine, and isoleucine degradation and biosynthesis pathways were significantly enriched in the -4 °C vs 4 °C and -4 °C vs 0 °C groups. Under negative ion mode, galactose metabolism and glycerolipid metabolism were significantly enriched in the 4 °C vs 0 °C and -4 °C vs 4 °C groups (*P* < 0.05). This study systematically elucidates the effects of storage temperature on truffle preservation and its underlying mechanisms, providing a theoretical basis for postharvest freshness maintenance of truffles.

## Data Availability

The original contributions presented in the study are included in the article/**Supplementary Material**. Further inquiries can be directed to the corresponding author.
