# Supplementary material for: Metabolomics-based analysis of the effects of different storage temperatures on the post-harvest quality of truffles
Source: Front Plant Sci. 2026 Jul 23;17:1880094. doi: 10.3389/fpls.2026.1880094 (PMC13441833; doi:10.3389/fpls.2026.1880094)
Supplement: Supplementary file 1 [file Supplementaryfile1.docx]

Metabolomics-Based Analysis of the Effects of Different Storage Temperatures on the Post-Harvest Quality of Truffles

Yuan Qing^a,b^ *, Junhong Jiang^a,b^ , Yao Wang^a,b^ , Qiang Li ^c^, Deju Yang^a,b^ , Siying Wu^a,b^ , Xiaoyin Dan^a,b^ , Hai Li^a,b^

a College of Agricultural Sciences, Xichang University, Xichang 615000, China;

b Panxi Crop Improvement Key Laboratory of Sichuan Province, Xichang University, Xichang 615000, China;

c School of Food and Biological Engineering, Chengdu University, Chengdu 610106, China;

*Corresponding author:

Yuan Qing (yuanq@xcc.edu.cn)

Phone: 86-18981532725

* Present address: North Campus of Xichang University, North Industrial Park, Xichang, Sichuan Province, 615000, China.

Table S1 Differences in Metabolite Expression in Tuberous Fungi at Different Temperatures

| Compared Samples | Num. of Total Sig. | Num. of Sig.Up | Num. of Sig.down |
| --- | --- | --- | --- |
| 4℃vs0℃ neg | 10 | 3 | 7 |
| 4℃vs0℃ pos | 30 | 15 | 15 |
| -4℃vs0℃ neg | 13 | 7 | 3 |
| -4℃vs0℃ pos | 16 | 12 | 4 |
| -4℃vs4℃ neg | 37 | 18 | 19 |
| -4℃vs4℃ pos | 44 | 22 | 22 |

Table S2 Two-Factor ANOVA of Rottenness rate, Storage Time, and Storage Temperature

| Levene's Test for Homogeneity of Variances^a^ | | | |
| --- | --- | --- | --- |
| Dependent variable: Value | | | |
| F | df1 | df2 | Sig. |
| 3.663 | 22 | 46 | 0.000 |
| Test the null hypothesis that the variances of the errors of the dependent variable are equal across all groups. | | | |
| a. Design : Intercept + Temperature + Time + Temperature * Time | | | |

| Testing for Inter-Subject Effects | | | | | |
| --- | --- | --- | --- | --- | --- |
| Dependent variable: Value | | | | | |
| Source | Type III Sum of Squares | df | Mean Square | F | Sig. |
| Model Calibration | 39187.041^a^ | 22 | 1781.229 | 193.533 | .000 |
| Intercept | 14191.125 | 1 | 14191.125 | 1541.888 | .000 |
| Temperature | 16561.950 | 3 | 5520.650 | 599.827 | .000 |
| Time | 9526.425 | 5 | 1905.285 | 207.012 | .000 |
| Temperature * Time | 14440.343 | 14 | 1031.453 | 112.069 | .000 |
| Error | 423.372 | 46 | 9.204 |  |  |
| Total | 51447.049 | 69 |  |  |  |
| Total Corrections | 39610.412 | 68 |  |  |  |
| a. R^2^ =0.989（Adjust R^2^ = 0.984） | | | | | |

Table S3 Two-Factor ANOVA of Relative Electrical Conductivity, Storage Time, and Storage Temperature

| Levene's Test for Homogeneity of Variances^a^ | | | |
| --- | --- | --- | --- |
| Dependent variable: Value | | | |
| F | df1 | df2 | Sig. |
| 3.663 | 22 | 46 | .000 |
| Test the null hypothesis that the variances of the errors of the dependent variable are equal across all groups. | | | |
| a. Design : Intercept + Temperature + Time + Temperature * Time | | | |

| Testing for Inter-Subject Effects | | | | | |
| --- | --- | --- | --- | --- | --- |
| Dependent variable: Value | | | | | |
| Source | Type III Sum of Squares | df | Mean Square | F | Sig. |
| Model Calibration | 39187.041^a^ | 22 | 1781.229 | 193.533 | .000 |
| Intercept | 14191.125 | 1 | 14191.125 | 1541.888 | .000 |
| Temperature | 16561.950 | 3 | 5520.650 | 599.827 | .000 |
| Time | 9526.425 | 5 | 1905.285 | 207.012 | .000 |
| Temperature * Time | 14440.343 | 14 | 1031.453 | 112.069 | .000 |
| Error | 423.372 | 46 | 9.204 |  |  |
| Total | 51447.049 | 69 |  |  |  |
| Total Corrections | 39610.412 | 68 |  |  |  |
| a. R^2^ =0 .989（Adjust R^2^ =0.984） | | | | | |

Table S4 Two-Factor Analysis of Variance for Respiratory Rates, Storage Time, and Storage Temperature

| Levene's Test for Homogeneity of Variances^a^ | | | |
| --- | --- | --- | --- |
| Dependent variable: Value | | | |
| F | df1 | df2 | Sig. |
| 3.424 | 21 | 44 | .000 |
| Test the null hypothesis that the variances of the errors of the dependent variable are equal across all groups. | | | |
| a. Design : Intercept + Temperature + Time + Temperature * Time | | | |

| Testing for Inter-Subject Effects | | | | | |
| --- | --- | --- | --- | --- | --- |
| Dependent variable: Value | | | | | |
| Source | Type III Sum of Squares | df | Mean Square | F | Sig. |
| Model Calibration | 987134.692^a^ | 21 | 47006.414 | 80.806 | .000 |
| Intercept | 4234067.140 | 1 | 4234067.140 | 7278.509 | .000 |
| Temperature | 517313.703 | 3 | 172437.901 | 296.427 | .000 |
| Time | 66170.936 | 5 | 13234.187 | 22.750 | .000 |
| Temperature * Time | 411060.441 | 13 | 31620.034 | 54.356 | .000 |
| Error | 25595.759 | 44 | 581.722 |  |  |
| Total | 5214429.364 | 66 |  |  |  |
| Total Corrections | 1012730.450 | 65 |  |  |  |
| a. R^2^ = .975（Adjust R^2^ =0.963） | | | | | |

Table S5 Two-Factor Analysis of Variance for MDA Content, Storage Time, and Storage Temperature

| Levene's Test for Homogeneity of Variances^a^ | | | |
| --- | --- | --- | --- |
| Dependent variable: Value | | | |
| F | df1 | df2 | Sig. |
| 4.327 | 21 | 66 | .000 |
| Test the null hypothesis that the variances of the errors of the dependent variable are equal across all groups.。 | | | |
| a. Design : Intercept + Temperature + Time + Temperature * Time | | | |

| Testing for Inter-Subject Effects | | | | | |
| --- | --- | --- | --- | --- | --- |
| Dependent variable: Value | | | | | |
| Source | Type III Sum of Squares | df | Mean Square | F | Sig. |
| Model Calibration | 50.253^a^ | 21 | 2.393 | 23.398 | .000 |
| Intercept | 432.076 | 1 | 432.076 | 4224.679 | .000 |
| Temperature | 21.865 | 3 | 7.288 | 71.263 | .000 |
| Time | 11.197 | 5 | 2.239 | 21.896 | .000 |
| Temperature * Time | 17.225 | 13 | 1.325 | 12.955 | .000 |
| Error | 6.750 | 66 | .102 |  |  |
| Total | 501.085 | 88 |  |  |  |
| Total Corrections | 57.003 | 87 |  |  |  |
| a. R^2^ =0.882（Adjust R^2^ =0.844） | | | | | |

Table S6 Two-Factor Analysis of Variance for SDH Content, Storage Time, and Storage Temperature

| Levene's Test for Homogeneity of Variances^a^ | | | |
| --- | --- | --- | --- |
| Dependent variable: Value | | | |
| F | df1 | df2 | Sig. |
| 2.278 | 21 | 44 | .011 |
| Test the null hypothesis that the variances of the errors of the dependent variable are equal across all groups. | | | |
| a. Design : Intercept + Temperature + Time + Temperature * Time | | | |

| Testing for Inter-Subject Effects | | | | | |
| --- | --- | --- | --- | --- | --- |
| Dependent variable: Value | | | | | |
| Source | Type III Sum of Squares | df | Mean Square | F | Sig. |
| Model Calibration | 41901.570^a^ | 21 | 1995.313 | 114.338 | .000 |
| Intercept | 567130.874 | 1 | 567130.874 | 32498.486 | .000 |
| Temperature | 29583.893 | 3 | 9861.298 | 565.085 | .000 |
| Time | 2473.023 | 5 | 494.605 | 28.342 | .000 |
| Temperature * Time | 8913.504 | 13 | 685.654 | 39.290 | .000 |
| Error | 767.844 | 44 | 17.451 |  |  |
| Total | 613109.162 | 66 |  |  |  |
| Total Corrections | 42669.414 | 65 |  |  |  |
| a. R^2^ =0.982（Adjust R^2^ =0.973） | | | | | |

Table S7 Two-Factor Analysis of Variance for CAT Content, Storage Time, and Storage Temperature

| Levene's Test for Homogeneity of Variances^a^ | | | |
| --- | --- | --- | --- |
| Dependent variable: Value | | | |
| F | df1 | df2 | Sig. |
| 3.429 | 23 | 48 | .000 |
| Test the null hypothesis that the variances of the errors of the dependent variable are equal across all groups. | | | |
| a. Design : Intercept + Temperature + Time + Temperature * Time | | | |

| Testing for Inter-Subject Effects | | | | | |
| --- | --- | --- | --- | --- | --- |
| Dependent variable: Value | | | | | |
| Source | Type III Sum of Squares | df | Mean Square | F | Sig. |
| Model Calibration | 1466504.586^a^ | 23 | 63761.069 | 9.500 | .000 |
| Intercept | 14514890.883 | 1 | 14514890.883 | 2162.578 | .000 |
| Temperature | 356834.960 | 3 | 118944.987 | 17.722 | .000 |
| Time | 624228.081 | 5 | 124845.616 | 18.601 | .000 |
| Temperature * Time | 485441.544 | 15 | 32362.770 | 4.822 | .000 |
| Error | 322168.671 | 48 | 6711.847 |  |  |
| Total | 16303564.139 | 72 |  |  |  |
| Total Corrections | 1788673.256 | 71 |  |  |  |
| a. R^2^ =0.820（Adjust R^2^ =0.734） | | | | | |

Figure S1 Class I Metabolite Classification

Fig S2 PCA plot of the cationic metabolome of truffles at different storage temperatures

Fig S3 PCA Plot of Metabolomic Negative Ions in Truffles at Different Storage Temperatures
